# Supplementary material for: Circulating microRNAs Suggest Networks Associated with Biological Functions in Aggressive Refractory Type 2 Celiac Disease
Source: Biomedicines. 2022 Jun 14;10(6):1408. doi: 10.3390/biomedicines10061408 (PMC9219665; doi:10.3390/biomedicines10061408)
Supplement: Supplementary file 1 [file biomedicines-10-01408-s001.zip › Supplementary materials/Table S1.pdf]

Table S1. Patient description.

Cohort of CD patients	Sex	Age (Y)	Age at CD diagnosis (Y)	Age at RCD diagnosis (Y)	Duodenal histology	TG2 antibodies	Aberrant IEL (%)	Comorbidities	TCRγ	Therapy	GIP results
GFD-responsive	F	43	5	na	0	negative	na	hypothyroidism	na	na	negative
GFD-responsive	F	39	4	na	0	negative	na	none	na	na	negative
GFD-responsive	F	29	3	na	0	negative	na	hypothyroidism, diabetes type 1	na	na	negative
GFD-responsive	M	58	3	na	0	negative	na	none	na	na	negative
GFD-responsive	F	43	31	na	0	negative	na	none	na	na	negative
RCD1	F	34	26	27	3b	negative	<20%	none	policlonal	azatioprin	negative
RCD1	M	55	51	52	3c	positive	<20%	none	policlonal	budesonide	negative
RCD1	M	45	32	35	3b	negative	<20%	none	policlonal	azatioprin	negative
RCD1	F	64	53	58	3b	positive	<20%	autoimmune hepatites	policlonal	budesonide	negative
RCD1	F	47	43	44	3a	negative	<20%	none	policlonal	budesonide	negative
RCD1	F	43	32	40	3b	positive	<20%	thyroiditis	policlonal	budesonide	negative
RCD2	M	79	64	64	3c	negative	>20%	autoimmune hepatites, psoriasis, thyroiditis	monoclonal	cladibrin	negative
RCD2	F	38	32	33	3c	negative	>20%	autoimmune hepatites, thyroiditis, psoriasis, vasculitis	monoclonal	cladibrin	negative
RCD2	M	56	51	52	3c	negative	>20%	none	monoclonal	cladibrin	negative
RCD2	F	68	62	63	3c	negative	>20%	none	monoclonal	cladibrin	negative
RCD2	F	73	66	66	3c	positive	>20%	thyroiditis, autoimmune hepatites	monoclonal	cladibrin	negative
