# Supplementary material for: Circulating microRNAs Suggest Networks Associated with Biological Functions in Aggressive Refractory Type 2 Celiac Disease
Source: Biomedicines. 2022 Jun 14;10(6):1408. doi: 10.3390/biomedicines10061408 (PMC9219665; doi:10.3390/biomedicines10061408)
Supplement: Supplementary file 1 [file biomedicines-10-01408-s001.zip › Supplementary materials/Table S2.pdf]

Table S2. Dysregulated miRNAs in serum samples obtained from unresponsive subjects and subjects responsive to a gluten-free diet.

MiRNA name	Fold change, unresponsive vs. responsive	FDR adjusted *p* value
hsa-miR-27b-3p	6.73	0.00
hsa-miR-206	3.29	0.04
hsa-miR-216b-5p	2.62	0.03
hsa-miR-4536-5p	1.98	0.03
hsa-miR-548g-3p	1.82	0.04
hsa-miR-1245a	–1.23	0.02
hsa-miR-1269b	–1.23	0.02
hsa-miR-519c-3p	–1.23	0.02
hsa-miR-10a-5p	–1.24	0.04
hsa-miR-18b-5p	–1.24	0.04
hsa-miR-3130-3p	–1.24	0.04
hsa-miR-323a-5p	–1.24	0.04
hsa-miR-500a-5p+hsa-miR-501-5p	–1.24	0.04
hsa-miR-502-3p	–1.24	0.04
hsa-miR-514a-5p	–1.24	0.04
hsa-miR-127-5p	–1.25	0.02
hsa-miR-134-3p	–1.25	0.02
hsa-miR-144-3p	–1.25	0.02
hsa-miR-15a-5p	–1.25	0.02
hsa-miR-18a-5p	–1.25	0.02
hsa-miR-1908-3p	–1.25	0.02
hsa-miR-204-5p	–1.25	0.02
hsa-miR-219a-1-3p	–1.25	0.02
hsa-miR-299-3p	–1.25	0.02
hsa-miR-300	–1.25	0.02
hsa-miR-30a-3p	–1.25	0.02
hsa-miR-323b-5p	–1.25	0.02
hsa-miR-331-5p	–1.25	0.02
hsa-miR-378c	–1.25	0.02
hsa-miR-3918	–1.25	0.02
hsa-miR-422a	–1.25	0.02
hsa-miR-539-5p	–1.25	0.02
hsa-miR-553	–1.25	0.02
hsa-miR-663a	–1.25	0.02
hsa-let-7e-5p	–1.26	0.02
hsa-let-7g-5p	–1.26	0.02
hsa-miR-153-3p	–1.26	0.02
hsa-miR-205-5p	–1.26	0.02
hsa-miR-210-3p	–1.26	0.02
hsa-miR-5010-5p	–1.26	0.03
hsa-miR-512-3p	–1.26	0.03
hsa-miR-519e-3p	–1.26	0.03
hsa-miR-1250-5p	–1.27	0.02
hsa-miR-433-3p	–1.28	0.01
hsa-miR-572	–1.29	0.02
hsa-let-7f-5p	–1.29	0.02
hsa-miR-1-3p	–1.29	0.02
hsa-miR-106a-5p+hsa-miR-17-5p	–1.29	0.02
hsa-miR-1185-1-3p	–1.29	0.02

hsa-miR-1204	–1.29	0.02
hsa-miR-1224-3p	–1.29	0.02
hsa-miR-1224-5p	–1.29	0.02
hsa-miR-1233-3p	–1.29	0.02
hsa-miR-1247-5p	–1.29	0.02
hsa-miR-125a-5p	–1.29	0.02
hsa-miR-128-2-5p	–1.29	0.02
hsa-miR-1281	–1.29	0.02
hsa-miR-1285-3p	–1.29	0.02
hsa-miR-1288-3p	–1.29	0.02
hsa-miR-1291	–1.29	0.02
hsa-miR-1301-3p	–1.29	0.02
hsa-miR-130b-3p	–1.29	0.02
hsa-miR-133b	–1.29	0.02
hsa-miR-135a-5p	–1.29	0.02
hsa-miR-142-5p	–1.29	0.02
hsa-miR-1469	–1.29	0.02
hsa-miR-151a-5p	–1.29	0.02
hsa-miR-151b	–1.29	0.02
hsa-miR-152-3p	–1.29	0.02
hsa-miR-188-3p	–1.29	0.02
hsa-miR-1910-3p	–1.29	0.02
hsa-miR-193a-5p+hsa-miR-193b-5p	–1.29	0.02
hsa-miR-193b-3p	–1.29	0.02
hsa-miR-194-5p	–1.29	0.02
hsa-miR-1973	–1.29	0.02
hsa-miR-20a-5p+hsa-miR-20b-5p	–1.29	0.02
hsa-miR-211-3p	–1.29	0.02
hsa-miR-2110	–1.29	0.02
hsa-miR-221-5p	–1.29	0.02
hsa-miR-3065-5p	–1.29	0.02
hsa-miR-31-5p	–1.29	0.02
hsa-miR-3140-3p	–1.29	0.02
hsa-miR-3140-5p	–1.29	0.02
hsa-miR-3192-5p	–1.29	0.02
hsa-miR-3195	–1.29	0.02
hsa-miR-320b	–1.29	0.02
hsa-miR-326	–1.29	0.02
hsa-miR-328-3p	–1.29	0.02
hsa-miR-328-5p	–1.29	0.02
hsa-miR-335-5p	–1.29	0.02
hsa-miR-33a-5p	–1.29	0.02
hsa-miR-362-3p	–1.29	0.02
hsa-miR-365b-5p	–1.29	0.02
hsa-miR-369-5p	–1.29	0.02
hsa-miR-384	–1.29	0.02
hsa-miR-409-3p	–1.29	0.02
hsa-miR-409-5p	–1.29	0.02
hsa-miR-412-3p	–1.29	0.02
hsa-miR-425-5p	–1.29	0.02
hsa-miR-4485-3p	–1.29	0.02
hsa-miR-449a	–1.29	0.02

hsa-miR-455-3p	–1.29	0.02
hsa-miR-4787-3p	–1.29	0.02
hsa-miR-485-5p	–1.29	0.02
hsa-miR-487b-3p	–1.29	0.02
hsa-miR-490-5p	–1.29	0.02
hsa-miR-491-5p	–1.29	0.02
hsa-miR-494-5p	–1.29	0.02
hsa-miR-501-3p	–1.29	0.02
hsa-miR-504-3p	–1.29	0.02
hsa-miR-505-3p	–1.29	0.02
hsa-miR-508-5p	–1.29	0.02
hsa-miR-518d-3p	–1.29	0.02
hsa-miR-518e-3p	–1.29	0.02
hsa-miR-525-3p	–1.29	0.02
hsa-miR-532-5p	–1.29	0.02
hsa-miR-548ad-3p	–1.29	0.02
hsa-miR-550a-5p	–1.29	0.02
hsa-miR-574-3p	–1.29	0.02
hsa-miR-580-3p	–1.29	0.02
hsa-miR-590-3p	–1.29	0.02
hsa-miR-604	–1.29	0.02
hsa-miR-614	–1.29	0.02
hsa-miR-617	–1.29	0.02
hsa-miR-619-3p	–1.29	0.02
hsa-miR-639	–1.29	0.02
hsa-miR-641	–1.29	0.02
hsa-miR-6503-3p	–1.29	0.02
hsa-miR-664b-5p	–1.29	0.02
hsa-miR-708-5p	–1.29	0.02
hsa-miR-885-5p	–1.29	0.02
hsa-miR-887-3p	–1.29	0.02
hsa-miR-9-5p	–1.29	0.02
hsa-miR-934	–1.29	0.02
hsa-miR-937-3p	–1.29	0.02
hsa-miR-1260b	–1.31	0.02
hsa-miR-182-5p	–1.31	0.02
hsa-miR-376b-3p	–1.31	0.02
hsa-miR-4435	–1.31	0.02
hsa-miR-4792	–1.31	0.04
