# Supplementary material for: Circulating microRNAs Suggest Networks Associated with Biological Functions in Aggressive Refractory Type 2 Celiac Disease
Source: Biomedicines. 2022 Jun 14;10(6):1408. doi: 10.3390/biomedicines10061408 (PMC9219665; doi:10.3390/biomedicines10061408)
Supplement: Supplementary file 1 [file biomedicines-10-01408-s001.zip › Supplementary materials/Table S3.pdf]

Table S3. Main functional target genes common to several dysregulated miRNAs involved in biological processes and identified in serum from RCD2 patients.

MiRNAs	Gene	Name	Annotation	Validations, miRTarBase	Validations, TarBase (DIANA)	Ref
hsa-miR-101-3p (down)<br>hsa-miR-107 (down)	**ACVR2B**	Activin A Receptor Type 2B	GO:0071559	NGS	PAR-CLIP<br>HITS-CLIP<br>IP<br>RNase treatment<br>Mild MNase digestion<br>Treatment with arsenite	[17, 18]
hsa-let-7d-5p (down)<br>hsa-miR-107 (down)	**AGO4**	Argonaute RISC Component 4	GO:0040029<br>GO:0198738<br>GO:0010608			
hsa-miR-302c-3p (up)<br>hsa-miR-101-3p (down)	**ASAP1**	ArfGAP With SH3 Domain Ankyrin Repeat and PH Domain 1	GO:0043087		Microarrays<br>PAR-CLIP	[17, 19, 20]
hsa-miR-302c-3p (up)<br>hsa-miR-770-5p (up)	**BTG1**	BTG Anti-Proliferation Factor 1	GO:0001525	NGS<br>CLIP-seq	PAR-CLIP IP<br>T1 RNase treatment	[19]
hsa-miR-770-5p (up)<br>hsa-let-7d-5p (down)	**CCND2**	Cyclin D2	GO:0044772<br>GO:0071900			
hsa-let-7d-5p (down)<br>hsa-miR-181b-2-3p (up)	**CEP135**	Centrosomal protein 135	GO:0044772	NGS<br>CLIP -seq		[21]
hsa-miR-935 (down)<br>hsa-let-7d-5p (down)	**CPEB1**	Cytoplasmic Polyadenylation Element Binding Protein 1	GO:0001101<br>GO:0010608			
hsa-miR-101-3p (down)<br>hsa-miR-770-5p (up)<br>hsa-miR-107 (down)	**CPEB3**	Cytoplasmic Polyadenylation Element Binding Protein 3	GO:0001101<br>GO:0050769<br>GO:0010608		HITS-CLIP<br>PAR-CLIP<br>Mild MNase digestion, chimeric fragments	[17, 18, 22]
hsa-miR-101-3p (down)<br>hsa-miR-302c-3p (up)	**CREBRF**	CREB3 Regulatory Factor	GO:0048545	NGS<br>CLIP -seq		[23]
hsa-miR-935 (down)	**DMD**	Dystrophin	GO:0032409		PAR-CLIP<br>shRNAs	[24]

hsa-let-7d-5p (down)					against HIV-1	
hsa-miR-101-3p (down) hsa-miR-181b-2-3p (up)	**DR1**	Down-Regulator of Transcription 1	GO:0016569		IPs HITS-CLIP	[22, 25]
hsa-miR-302c-3p (up) hsa-miR-107 (down)	**ELK4**	ETS Transcription Factor ELK4	GO:0016569	NGS CLIP-seq	PAR-CLIP HITS-CLIP IP RNase treatment Mild MNase digestion Treatment with arsenite	[17-19, 25-29]
hsa-miR-320e (up) hsa-miR-107 (down)	**EPHA7**	EPH Receptor A7	GO:0002009 GO:0061564 GO:0090066		PAR-CLIP IP RNase treatment	[18]
hsa-miR-935 (down) hsa-miR-1226-3p (up) hsa-miR-107 (down)	**FBXW7**	F-box and WD Repeat domain containing 7	GO:0001525	Reporter assay Western blot microarray CLIP-seq	PAR-CLIP HITS-CLIP IP shRNAs against HIV-1 RNase treatment Arsenite treatment	[17, 18, 22-26, 28-30]
hsa-let-7d-5p (down) hsa-miR-302c-3p (up)	**FZD3**	Frizzled Class Receptor 3	GO:0198738 GO:0002009 GO:0050769 GO:0061564		PAR-CLIP shRNAs against HIV-1 Mild MNase digestion	[18, 24, 29]
hsa-miR-935 (down) hsa-miR-101-3p (down) hsa-miR-302c-3p (up)	**FZD6**	Frizzled Class Receptor 6	GO:0198738 GO:0002009	NGS, CLIP-seq	Chimeric fragments, MICROARRAYS HITS-CLIP PAR-CLIP Mild MNase digestion T1 RNase treatment	[17–19, 28, 31–33]
hsa-miR-490-3p (up) hsa-miR-107 (down)	**KDM7A**	Lysine Demethylase 7A	GO:0016569			
hsa-miR-101-3p (down)	**LRP2**	LDL receptor related	GO:0002009 GO:0050769			

hsa-miR-302c-3p (up)		protein 2				
hsa-miR-371a-5p (up)<br>hsa-miR-770-5p (up)	**MAP3K1**	Mitogen-Activated Protein Kinase Kinase Kinase 1	GO:0071900			
hsa-let-7d-5p (down)<br>hsa-miR-1306-5p (down)	**MAPK6**	Mitogen-Activated Protein Kinase 6	GO:0050769	NGS<br>CLIP-seq	PAR-CLIP<br>HITS-CLIP<br>IP<br>RNase treatment	[18, 25, 34]
hsa-miR-101-3p (down)<br>hsa-miR-770-5p (up)	**MARK1**	Microtubule Affinity Regulating Kinase 1	GO:0198738		HITS-CLIP	[22]
hsa-miR-101-3p (down)<br>hsa-miR-302c-3p (up)<br>hsa-miR-107 (down)	**MYCN**	MYCN proto-oncogene bHLH transcription factor	GO:0040029<br>GO:0010608	Reporter assay, Western blot, NGS, CLIP-seq	Luciferase reporter assay, chimeric fragments	[31, 35, 36]
hsa-miR-181b-2-3p (up)<br>hsa-miR-770-5p (up)	**MYH10**	Myosin Heavy Chain 10	GO:0001701<br>GO:0061564			
hsa-miR-935 (down)<br>hsa-let-7d-5p (down)	**ONECUT2**	One Cut Homeobox 2	GO:0071559	NGS, CLIP-seq		[23, 29]
hsa-let-7d-5p (down)<br>hsa-miR-770-5p (up)	**PBX1**	PBX homeobox 1	GO:0044772<br>GO:0002009		PAR-CLIP<br>shRNAs against HIV-1<br>HITS-CLIP	[24, 30]
hsa-miR-302c-3p (up)<br>hsa-miR-107 (down)	**PPP6C**	Protein Phosphatase 6 Catalytic subunit	GO:0044772<br>GO:0016311	NGS, CLIP-seq	PAR-CLIP<br>HITS-CLIP<br>Mild MNase digestion	[17, 18, 26, 29]
hsa-miR-302c-3p (up)<br>hsa-miR-1306-5p (down)	**RELA**	RELA proto-oncogen NF-kB subunit	GO:0001101<br>GO:0050769<br>GO:0048545<br>GO:1902532			
hsa-miR-935 (down)<br>hsa-miR-101-3p (down)	**RORA**	RAR Related Orphan Receptor A	GO:0001525<br>GO:0048545<br>GO:1902532	NGS<br>CLIP-seq	Microarray<br>PAR-CLIP IP<br>RNase treatment	[17-20, 26]
hsa-miR-101-3p (down)<br>hsa-miR-107 (down)	**SIX4**	SIX Homeobox 4	GO:0002009	NGS, CLIP-seq	PAR-CLIP<br>Mild MNase digestion	[18, 29]

hsa-miR-101-3p (down) hsa-miR-606 (down)	**SMARCA5**	SWI/SNF Related Matrix Associated Actin Dependent Regulator of Chromatin Subfamily A Member 5	GO:0040029		Microarrays HITS-CLIP	[20, 34]
hsa-miR-101-3p (down) hsa-miR-606 (down)	**STC1**	Stanniocalcin 1	GO:0048545	CLIP-seq	Microarrays	[32]
hsa-miR-320e (up) hsa-miR-107 (down)	**TDG**	Thymine DNA Glycosylase	GO:0040029			
hsa-miR-101-3p (down) hsa-miR-302c-3p (up)	**TET2**	Tet Methylcytosine Dioxygenase 2	GO:0016569		Luciferase reporter assay PAR-CLIP T1 RNase treatment	[19, 26, 37]
hsa-let-7d-5p (down) hsa-miR-302c-3p (up) hsa-miR-1306-5p (down)	**TET3**	Tet Methylcytosine Dioxygenase 3	GO:0016569		PAR-CLIP shRNAs against HIV-1 HITS-CLIP	[24, 27, 30]
hsa-let-7d-5p (down) hsa-miR-101-3p (down) hsa-miR-107 (down)	**TGFBR3**	Transforming Growth Factor β Receptor 3	GO:0071559 GO:0001101	NGS, CLIP-seq	PAR-CLIP Mild MNase digestion HITS-CLIP	[18, 33, 34]
hsa-miR-101-3p (down) hsa-miR-181b-2-3p (up) hsa-miR-107 (down)	**TNRC6B**	Trinucleotide Repeat Containing Adaptor 6B	GO:0040029 GO:0198738 GO:0010608		HITS-CLIP Microarrays	[20, 22, 33]
hsa-let-7d-5p (down) hsa-miR-107 (down)	**TRIM71**	Tripartite Motif Containing 71	GO:0040029 GO:0044772 GO:0002009 GO:0010608	NGS CLIP-seq	PAR-CLIP Mild MNase digestion	[18, 29]
hsa-miR-101-3p (down) hsa-miR-770-5p (up)	**UBR3**	Ubiquitin Protein Ligase E3 Component N-Recognin 3	GO:0001701		HITS-CLIP	[22]
hsa-miR-101-3p	**VLDLR**	Very Low	GO:0050769			

(down)<br>hsa-miR-302c-3p<br>(up)		Density<br>Lipoprotein<br>Receptor	GO:0061564			
